# Development and Evaluation of a Phospholipid Complex-Loaded SMEDDS for Enhanced Oral Delivery of H007, a Novel Anti-Hyperlipidemic Drug

**DOI:** 10.3390/pharmaceutics18040474

**Published:** 2026-04-13

**Authors:** Chunxi Liu, Lundang Guo, Liqing Chen, Xiaoliang Gong, Zunsheng Han, Jing Feng, Chi Zhang, Song Wu, Qingyun Yang

**Affiliations:** State Key Laboratory of Bioactive Substance and Function of Natural Medicines, Institute of Materia Medica, Chinese Academy of Medical Sciences & Peking Union Medical College, Beijing 100050, China; liuchunxi@imm.ac.cn (C.L.); guolundang@imm.ac.cn (L.G.); chenliqing@imm.ac.cn (L.C.); g1463419733@163.com (X.G.); hzs@imm.ac.cn (Z.H.); fengjing@imm.ac.cn (J.F.); zczhang@imm.ac.cn (C.Z.)

**Keywords:** H007, SMEDDS/self-microemulsifying drug delivery system, phospholipid complex, anti-hyperlipidemic, oral bioavailability

## Abstract

**Background**: H007 is a novel selective AMP-activated protein kinase (AMPK) activator with demonstrated efficacy against hyperlipidemia; however, its oral bioavailability is limited by poor solubility and low intestinal permeability. This study aimed to develop a self-microemulsifying drug delivery system (SMEDDS) incorporating a H007–phospholipid complex (H007-PC) to improve both solubility and intestinal permeability. **Methods**: H007-PC-SME was prepared by integrating phospholipid complexes into an SMEDDS formulation. The formulation was optimized on the basis of emulsification efficiency, droplet size, and zeta potential, and was then evaluated for stability, in vitro drug release, and cellular uptake. Different H007 formulations were orally administered to golden hamsters to assess bioavailability, and a chylomicron flow blockade hamster model was used to evaluate lymphatic transport. **Results**: The optimized H007-PC-SME showed good stability, rapid self-emulsification, and improved drug solubility. Relative to ordinary H007 tablets, the relative bioavailability of H007-SME and H007-PC-SME was 376.65% and 464.62%, respectively, when calculated from M1 exposure, and 314.01% and 463.55%, respectively, when calculated from MP exposure. When evaluated in a cycloheximide model, H007-SME and H007-PC-SME increased the lymphatic transport fraction of M1 from approximately 0% to 22% and 54%, and that of MP from approximately 1% to 28% and 52% compared with ordinary H007 tablets. **Conclusions**: H007-PC-SME combines stable phospholipid complex formation with strong self-emulsification performance and effective drug dissolution. By overcoming the intrinsic limitations of the H007 active pharmaceutical ingredient and ordinary H007 tablets, this formulation improves membrane permeability and lymphatic transport, thereby enhancing oral bioavailability and therapeutic potential. The formulation shows good stability and acceptable in vitro biocompatibility under the tested conditions. The preparation process is straightforward, reproducible, and suitable for further pharmaceutical development.

## 1. Introduction

Hyperlipidemia, characterized by elevated levels of low-density lipoprotein cholesterol (LDL-C), cholesterol, triglycerides (TG), and/or reduced levels of high-density lipoprotein cholesterol (HDL-C), has become a global public health problem with major consequences for cardiovascular health [1]. Recent estimates indicate that 39% of adults worldwide have hypercholesterolemia, with significant regional variation. The prevalence exceeds 50% in high-income countries, whereas low-income countries are experiencing an increasing burden of dyslipidemia driven by urbanization and dietary transition. Approximately 3.72 million deaths each year are directly attributable to elevated LDL-C, ranking it as the eighth leading global risk factor for mortality [2,3,4].

Current treatment strategies rely mainly on statins, which reduce the incidence of cardiovascular events but remain constrained by a 10–20% intolerance rate, including adverse reactions such as rhabdomyolysis and hepatotoxicity, and by poor patient adherence. These challenges are particularly evident in low- and middle-income countries (LMICs), where access to lipid-lowering therapies remains restricted. Even among patients with good adherence, 40–60% do not achieve target LDL-C levels [5,6,7,8], highlighting the need to identify and characterize novel agents capable of regulating key pathogenic pathways in hyperlipidemia [9].

H007 has emerged as a novel therapeutic candidate with distinctive pharmacological properties. It is a small-molecule AMP-activated protein kinase (AMPK) agonist with strong lipid-regulating activity [10,11,12,13], supported by extensive experimental evidence. Studies using a hyperlipidemic golden hamster model have demonstrated that H007 administration significantly reduces serum TG, total cholesterol (TC), and LDL-C levels [14,15,16]. Mechanistic investigations [17] further demonstrate that H007 acts as a selective activator of the AMPK pathway, suggesting multimodal regulatory effects on metabolic homeostasis. Together, these findings support the potential of H007 as an effective and low-toxicity anti-hyperlipidemic agent.

**Figure 1:**
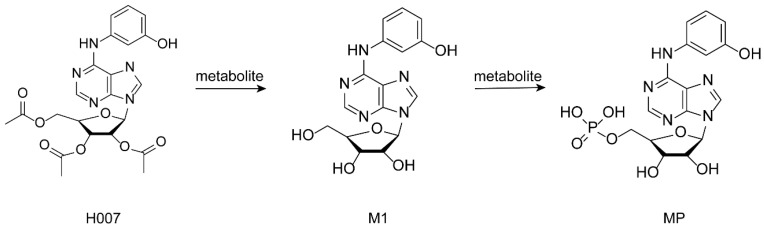
Molecular structures of H007 as the prodrug, M1, and MP as the active metabolites in vivo.

The pharmacological activity of H007 depends on sequential metabolic activation followed by pathway modulation. After systemic absorption, H007 is biotransformed to its metabolite M1, which is subsequently phosphorylated after cellular uptake to generate the active metabolite MP (Figure 1). At the molecular level, MP allosterically activates AMPK, suppresses de novo hepatic lipid synthesis, enhances hepatic lipid oxidation and utilization, regulates genes involved in fatty acid oxidation and gluconeogenesis, downregulates lipid synthesis-related genes, and activates the hepatic AMPK/ACC signaling pathway, thereby improving lipid metabolic disorders in vivo [18].

The poor aqueous solubility of H007 leads to low oral bioavailability and severely limits dosage-form development. Preclinical pharmacokinetic studies have shown that, after oral administration to golden hamsters, the bioavailability of H007 calculated from M1 ranged from 2.01% to 2.23%, whereas that calculated from MP ranged from 4.87% to 6.57%. In rhesus monkeys, the bioavailability calculated from M1 was 5.70–7.52%, whereas that calculated from MP was 1.04–1.11% [18]. For chronic disease treatment, low bioavailability necessitates higher doses and repeated administration, reducing patient compliance and limiting clinical application. Therefore, improving the oral bioavailability of H007 through formulation strategies has clear clinical relevance.

Self-microemulsifying drug delivery systems (SMEDDS) are isotropic mixtures of oil, surfactant, and co-surfactant that spontaneously disperse in gastrointestinal fluids to form microemulsions or nanoemulsions with nanoscale droplets [19]. The resulting droplet sizes, typically 10–100 nm, enhance dissolution rates and membrane permeability [20]. Compared with alternative delivery approaches, SMEDDS offer several advantages, including simple preparation, favorable thermodynamic stability, and low production cost. For poorly water-soluble drugs such as H007, lipophilicity is a key determinant of successful incorporation into SMEDDS [21]. Recent studies have shown that the formation of drug–phospholipid complexes can induce molecular reorganization and improve physicochemical properties and biological activity relative to the parent compound, thereby increasing drug lipophilicity and facilitating encapsulation into SMEDDS [22,23]. However, because of their increased lipophilicity, phospholipid complexes often show limited aqueous solubility and dispersibility [24,25]. Additional formulation strategies are therefore required to improve the dissolution behavior of the H007–phospholipid complex (H007-PC).

Therefore, the primary objective of this study was to develop an SMEDDS containing an H007-PC and to evaluate its performance using in vitro and in vivo approaches. An SMEDDS was systematically designed and optimized to identify excipients suitable for oral administration. Key properties, including morphology, particle size, polydispersity index (PDI), zeta potential, and formulation stability, were characterized. Cellular uptake was assessed using a Caco-2 cell model. In addition, comparative pharmacokinetic analysis and a hamster chylomicron flow blockade model were used to evaluate intestinal absorption and lymphatic transport of H007-PC-SME relative to H007-SME. Overall, the optimized H007-PC-SME formulation represents a promising strategy for improving the oral bioavailability of H007 and may support its further clinical development as an effective oral antihyperlipidemic therapy.

## 2. Materials and Methods

### 2.1. Materials

H007, with a purity of 99.87% as assessed by high-performance liquid chromatography (HPLC), and ordinary H007 tablets (150 mg per tablet, containing 100 mg H007) were obtained from the Institute of Materia Medica, Chinese Academy of Medical Sciences (CAMS) (Beijing, China). Lecithin (phosphatidylcholine, PC ≥ 80%) and medium chain triglycerides (MCT) were purchased from Shanghai Yuanye Biological Technology Co., Ltd. (Shanghai, China). Kolliphor^®^ HS15 and polyethylene glycol 400 were purchased from Beijing Fengli Jingqiu Pharmaceutical Co., Ltd. (Beijing, China). Capmul^®^ MCM and Labrasol were purchased from Shanghai Aladdin Biochemical Technology Co., Ltd. (Shanghai, China). 4′,6-diamidino-2-phenylindole (DAPI) and Cell-counting kit-8 (CCK-8) were purchased from Shanghai Beyotime Biotechnology Co., Ltd. (Shanghai, China). Anti-fluorescence fading mounting medium was purchased from Wuhan Yilerite Biotechnology Co., Ltd. (Wuhan, China). Coumarin 6 (Cou-6) was obtained from Beijing Konoscience Technology Co., Ltd. (Beijing, China).

### 2.2. Preparation and Characterization of H007–Phospholipid Complex

#### 2.2.1. Preparation of H007–Phospholipid Complex

H007-PC was prepared using a solvent evaporation method at a mass ratio of H007 to phospholipid of 1:0.67 (*w*/*w*). Briefly, 1.0 g of H007 and 0.67 g of phospholipid were placed in a 50 mL round-bottom flask and dissolved in 15 mL of chloroform at 40 °C. The mixture was stirred for 2 h, and the solvent was then removed under reduced pressure using a rotary evaporator at 45 °C. The resulting residue was collected and dried under vacuum.

#### 2.2.2. Infrared Spectral Analysis

Fourier transform infrared (FT-IR) spectra of H007, PC, H007-PC, and the physical mixture (PM) of H007 and phospholipid were recorded using an FT-IR spectrometer (Nicolet is50, Thermo Fisher Scientific, Waltham, MA, USA) over the wavenumber range of 500–4000 cm^−1^. Samples were prepared as potassium bromide (KBr) pellets before analysis.

#### 2.2.3. Differential Scanning Calorimetry (DSC) Analysis

Thermal behavior was examined by differential scanning calorimetry using a DSC system (DSC1 STAR^e^, Mettler-Toledo, Greifensee, Switzerland). H007, PC, H007-PC, and PM were sealed in aluminum pans and heated from 25 °C to 250 °C at a rate of 10 °C/min under a continuous nitrogen flow of 40 mL/min.

#### 2.2.4. X-Ray Powder Diffraction (XRPD) Analysis

XRPD patterns were collected using a D8 ADVANCE diffractometer (Rigaku Ultima IV, Tokyo, Japan) with Cu-Kα radiation operated at 40 kV and 40 mA. Data were acquired over a 2θ range of 3–80°, with a step size of 0.02° and a scanning speed of 8°/min.

### 2.3. Preparation of SMEDDS

#### 2.3.1. Solubility Study for Selection of Excipients for SMEDDS

The solubility of H007-PC in various excipients was determined using an equilibrium method [26]. Excess H007-PC was added to tubes containing 1 g of oils (Capmul^®^ MCM, MCT, oleic acid, and isopropyl myristate), surfactants (Labrasol, Tween 80, Kolliphor^®^ HS15, and Cremophor EL), or cosurfactants (polyethylene glycol 400, Transcutol P, anhydrous ethanol, and isopropyl alcohol). The mixtures were vortexed to ensure dispersion and then sonicated in a water bath for 30 min. Samples were then equilibrated in an air-bath shaker at 25 °C and 300 rpm for 48 h to allow complete precipitation of undissolved H007-PC. After equilibration, the samples were centrifuged at 15,000× *g* for 10 min. The supernatants were diluted with acetonitrile, and the concentration of H007-PC was determined by HPLC.

#### 2.3.2. Compatibility Test

On the basis of solubility results, MCT, Kolliphor^®^ HS15, and anhydrous ethanol were selected as the oil phase, surfactant and cosurfactant, respectively.

The oil phase and surfactant were mixed at mass ratios ranging from 1:9 to 5:5, while the total mass was maintained at 1 g. After thorough mixing, 100 μL of each formulation was added to 10 mL of distilled water to initiate self-emulsification. The resulting dispersions were evaluated according to established self-emulsification grading criteria, and their physical appearance and grades were recorded.

For compatibility evaluation, the surfactant and cosurfactant were weighed in equimolar amounts and mixed by vortexing to obtain a homogeneous system. The mixture was then centrifuged at 15,000× *g* for 10 min [27]. Phase separation after centrifugation was used as the criterion for compatibility. Systems without phase separation were considered compatible, whereas those showing stratification were considered incompatible.

#### 2.3.3. Evaluation of Emulsification and Phase Separation

SMEDDS was added dropwise to purified water at a ratio of 1:100 (*v*/*v*) under magnetic stirring at 37.0 ± 0.5 °C. The emulsification process and the resulting dispersion were visually assessed using a modified Khoo classification system (Table 1) [28]. Self-emulsifying performance was graded as follows. Grade I indicated a transparent or pale-blue microemulsion formed rapidly within 1 min. Grade II indicated a deep white emulsion formed rapidly within 1 min, with a milk-like appearance. Grade III indicated a milky emulsion. Grade IV indicated a slightly oily, grayish-white emulsion. Grade V indicated poor or almost no emulsification, with large oil droplets visible on the surface.

#### 2.3.4. Pseudo-Ternary Phase Diagram

To determine the self-emulsifying region of the SMEDDS, a series of formulations was systematically evaluated. A pseudo-ternary phase diagram was constructed to identify the optimal concentration ranges of the formulation components [29]. The diagram was generated by stepwise addition of the aqueous phase using a water titration method. Surfactants and cosurfactants were first mixed at different weight ratios (Km: 4:1, 2:1, 3:2, 1:1, 2:3, 1:2, and 1:4), followed by blending with MCT at oil-phase ratios ranging from 1:9 to 5:5 relative to the surfactant/co-surfactant mixture.

After homogenization, 100 μL of blank SME formulation was added to 10 mL of purified water under gentle magnetic stirring. Self-emulsification behavior was visually evaluated. Formulations that produced clear, transparent dispersions or showing slight blue opalescence were considered acceptable and classified within the A and B grading range. The data were plotted using Origin software (2021, Northampton, MA, USA), and the region corresponding to efficient self-emulsification was quantified to generate the final pseudo-ternary phase diagram.

#### 2.3.5. Experimental Optimization of the H007-PC-SME Formulation

The final formulation was optimized using central composite design (CCD) coupled with response surface methodology. Previous studies have indicated that oil content and Km are critical factors affecting SMEDDS performance [30]. Because the pseudo-ternary phase diagram defines only the feasible formulation range, further optimization was required. As shown in Table 2, a star-point response surface design was applied using a two-factor, five-level experimental scheme for SMEDDS containing H007-PC. Based on preliminary screening, oil percentage (X_1_) and Km (X_2_) were selected as independent variables, whereas droplet size (Y_1_), PDI (Y_2_), and equilibrium solubility (Y_3_) were selected as dependent variables. Experimental data were analyzed by nonlinear regression using Design-Expert software version 13.0 (Stat-Ease Inc., Minneapolis, MN, USA). The best-fitting model was then used to generate contour plots and response-surface maps to predict the effects of the independent variables on formulation performance.

#### 2.3.6. Preparation of H007-SME and H007-PC-SME

According to the final optimized formulation, weigh 11% (*w*/*v*) MCT as the oil phase, 65% (*w*/*v*) Kolliphor^®^ HS15 as the surfactant, and 24% (*w*/*v*) anhydrous ethanol as the co-surfactant into a vial. Add H007 or H007-PC (containing H007 50 mg/g) and mix thoroughly on a stirrer until homogeneous.

### 2.4. Determination of Droplet Size, PDI and Zeta Potential

Droplet size, PDI, and zeta potential of H007-PC-SME after dilution with water were measured immediately following the self-emulsification study. Droplet size and PDI were determined by dynamic light scattering based on laser scattering principles, whereas zeta potential was measured using electrophoretic light scattering (pH 4.6). All measurements were performed in triplicate [31,32].

### 2.5. Morphological Analysis by Transmission Electron Microscopy

H007-SME and H007-PC-SME were diluted with water at a ratio of 1:100 to form microemulsions. A drop of each dispersion was placed onto a carbon-coated copper grid and allowed to stand for 5 min. Excess liquid was removed with filter paper, followed by negative staining with phosphotungstic acid for 5 min. After removal of excess stain, samples were dried under an infrared lamp for 5 min. The dried grids were examined using a transmission electron microscope (TEM; HT7800, Hitachi, Tokyo, Japan) [33].

### 2.6. Effect of pH and Dilution Stability

H007-PC-SME was diluted with distilled water and buffer solutions at pH 3.8, pH 4.5, and pH 6.8 to dilution factors of 50, 100, 250, 500 and 1000. The diluted systems were stored for 24 h and then visually inspected for clarity, drug precipitation, and phase separation.

### 2.7. Stability Test

A preliminary stability assessment was conducted under stress conditions, including elevated temperature (60 °C), high humidity (25 °C, RH 90% ± 5% and 25 °C, RH 75% ± 5%), and strong light exposure (4500 ± 500 lx). At predefined time points (days 5, 10, and 30), samples were evaluated for changes in appearance, droplet size, PDI, zeta potential and H007 content. All parameters were compared with baseline values recorded on day 0.

### 2.8. HPLC Analysis

The content of H007 in H007-SME and H007-PC-SME was quantified using an HPLC system (LC-10AT, Shimadzu, Kyoto, Japan) equipped with a Waters Symmetry C18 column (250 × 4.6 mm, 5 μm) maintained at 30 °C. The mobile phase consisted of acetonitrile and distilled water (35: 65, *v*/*v*) containing 0.2% acetic acid. The flow rate was set at 1.0 mL/min. A 10 μL sample was injected, and detection was performed at 300 nm.

### 2.9. In Vitro Drug Release

H007-PC-SME is an oily liquid formulation with a drug loading of 50 mg/g. It was filled into the largest available capsules to produce H007-PC-SME capsules (600 mg per capsule, containing 30 mg of H007), which were used as the test group. H007 tablets (150 mg per tablet, containing 100 mg of H007) were used as the control group. Dissolution studies were performed according to the Chinese Pharmacopoeia (2020 Edition, Part IV, General Rule 0931). The paddle method (Method II) was used for tablets, and the basket method (Method I) was used for capsules. Dissolution media included distilled water and buffer solutions at pH 3.8, pH 4.5, and pH 6.8 (1000 mL for ordinary H007 tablets, 500 mL for H007-PC-SME capsules), maintained at 37 ± 0.5 °C with a rotation speed of 75 rpm. Samples were collected at 5, 10, 15, 30, 45, and 60 min, and H007 concentrations were determined by HPLC–UV at 300 nm to compare dissolution profiles and cumulative drug release percentages between ordinary H007 tablets and H007-PC-SME capsules.

### 2.10. Cell Studies

The cytotoxicity of the formulations toward Caco-2 cells was evaluated using the CCK-8 assay. Briefly, Caco-2 cells were seeded into 96-well plates at a density of 1 × 10^4^ cells per well in 200 μL of culture medium and incubated for 24 h to allow cell attachment. The cells were then treated with H007 or H007-PC-SME at concentrations 0.1, 0.5, 1, 2, 5, 10, 15, and 25 μg/mL, while untreated cells served as the control. Each concentration was tested in eight replicate wells. After 24 h of incubation, the culture medium was removed, and 100 μL of CCK-8 solution (10% *v*/*v* in culture medium) was added to each well. The plates were incubated at 37 °C for an additional 2 h. Absorbance was measured at 450 nm using a microplate reader (Synergy H1, BioTek, Seattle, WA, USA). Cell viability was calculated using the following equation.

$$Cell\;viability(\%)=\frac{{OD}_{sample}-{OD}_{blank}}{{OD}_{control}-{OD}_{blank}}\times 100$$

#### Cellular Uptake Study

The cellular uptake efficiency of H007-PC-SME in Caco-2 cells was quantitatively assessed by flow cytometry (FCM). The hydrophobic fluorescent probe Cou-6 was incorporated into H007-SME and H007-PC-SME formulations. Caco-2 cells (2 × 10^5^ cells/well, in 1 mL) were seeded into 12-well plates and incubated at 37 °C for 24 h. The cells were then treated with free Cou-6 solution, Cou-6-SME, or Cou-6-PC-SME, at a final Cou-6 concentration of 1 μg/mL. After incubation for 4 h, the cells were washed three times with phosphate-buffered saline (PBS), detached using 0.25% trypsin, and centrifuged at 550× *g* for 5 min. The cell pellets were resuspended in PBS, passed through a 300-mesh filter, and analyzed using an FCM system (LongCyte™, Challenbio, Beijing, China).

Qualitative cellular uptake was further examined by laser confocal scanning microscopy (LCSM). Caco-2 cells (2 × 10^5^ cells/well in 1 mL) were seeded onto coverslips placed in 12-well plates and incubated at 37 °C for 24 h. The cells were treated with free Cou-6 solution, Cou-6-SME, or Cou-6-PC-SME at a final Cou-6 concentration of 1 μg/mL. After 4 h of incubation, the cells were washed three times with PBS and fixed with 1 mL of 4% paraformaldehyde for 10 min. Cell nuclei were stained with 400 μL of DAPI for 5 min in the dark. After two additional washes with cold PBS, the coverslips were examined using an LCSM system (TCS SP2, Leica, Wetzlar, Germany).

### 2.11. In Vivo Pharmacokinetic Studies

The lipoprotein profile of golden hamsters closely resembles that of humans. In addition, studies of H007 stability and metabolite identification of H007 in different animal species and in human liver microsomes indicated that the metabolite profiles in golden hamsters and human liver microsomes are consistent, with similar formation rates and amounts of the primary metabolite M1. Based on these considerations, golden hamsters were selected as the animal model for non-clinical pharmacokinetic evaluation of H007.

Golden hamsters (90 ± 10 g) were obtained from Beijing Vital River Laboratory Animal Technology Co., Ltd. (Beijing, China). All animal experiments were conducted in accordance with the NIH Guide for the Care and Use of Laboratory Animals and were approved by the Laboratory Animal Ethics Committee of the Institute of Materia Medica, CAMS. Pharmacokinetic studies were performed for ordinary H007 tablets, H007-PC, H007-SME, and H007-PC-SME using equal numbers of male and female animals. Before dosing, the hamsters were fasted for 12 h with free access to water. Twenty-four animals were randomly divided into four groups. Each group received a single oral dose of H007 at 70 mg/kg in a total volume of 0.7 mL by oral gavage. The control group received a suspension of ordinary H007 tablets, whereas the other groups received H007-PC, H007-SME, or H007-PC-SME. ordinary H007 tablets and H007-PC were suspended in 0.3% sodium carboxymethyl cellulose (CMC-Na) solution and finely ground to ensure dose uniformity. Blood samples (approximately 0.5 mL) were collected from the retro-orbital venous plexus at 0.25, 0.5, 0.75, 1, 2, 5, 8, 12, and 24 h after administration and immediately transferred into heparinized tubes.

### 2.12. Chylomicron Flow Blockade Hamster Model

To assess the lymphatic transport of H007 from H007-SME and H007-PC-SME in vivo, a chylomicron flow blockade model was applied under the same housing conditions described above. Cycloheximide (CHX) was administered intraperitoneally at a dose of 5.0 mg/kg in saline (0.83 mg/mL, *w*/*v*). One hour after cycloheximide administration, the animals received a single oral dose of H007 (70.0 mg/kg) as ordinary H007 tablets, H007-SME, or H007-PC-SME. Blood samples (approximately 0.5 mL) were collected via the retro-orbital sinus at 0.25, 0.5, 0.75, 1, 2, 5, 8, 12, and 24 h after dosing.

### 2.13. In Vivo Analysis

#### 2.13.1. Sample Handling

Plasma (50 μL) was transferred into a 1.5 mL conical centrifuge tube. Subsequently, 100 μL of acetonitrile working solution containing the internal standard (50 ng/mL) was added, and the mixture was vortex-mixed for 60 s. Samples were centrifuged at 13,000× *g* for 10 min. An aliquot of 50 μL of the supernatant was collected for injection, and plasma concentrations of H007 were determined under the chromatographic and mass spectrometric conditions described below.

#### 2.13.2. LC-MS/MS Analysis

The LC–MS/MS system consisted of an Agilent 1290 Infinity II HPLC (Agilent Technologies, Santa Clara, CA, USA) equipped with a vacuum degasser, binary pump, and autosampler, coupled to an Agilent 6495 LC/TQ triple quadrupole mass spectrometer (Agilent Technologies, Santa Clara, CA, USA) with an electrospray ionization (ESI) source.

Chromatographic conditions: Separation was performed on a ReproSil-Pur 120 C18-AQ column (3 μm, 2 mm × 100 mm). The injection volume was 2 μL, and the column temperature was maintained at 40 °C. Mobile phase A consisted of 0.1% (*v*/*v*) formic acid in acetonitrile, and mobile phase B consisted of 0.1% (*v*/*v*) ammonium formate with 0.1% (*v*/*v*) ammonia in water. The flow rate was set at 0.3 mL/min, and linear gradient elution was applied as described in Table 3.

Mass spectrometric conditions: Detection was performed using ESI in the positive-ion mode with multiple reaction monitoring (MRM). The nebulizer pressure was 20 psi, the nitrogen drying gas flow was 14 L/min at 200 °C, the sheath gas flow was 11 L/min at 250 °C, and the capillary voltage was set to 4000 V for the positive mode and 3500 V for the negative mode. The MRM transitions used for quantification were as follows: H007, [M+H]^+^ 486.0→228.0; M1, [M+H]^+^ 360.0→228.1; MP, [M+H]^+^ 440.0→228.1; internal standard (WS070119), [M+H]^+^ 374.0→136.1.

### 2.14. Statistical Analysis

Data are presented as mean ± standard deviation (SD). Statistical analyses were performed using GraphPad Prism 8.4 (GraphPad Software Inc., La Jolla, CA, USA). Pharmacokinetic parameters were calculated using DAS software (Drug and Statistics 2.0, Beijing, China) with a non-compartmental model analysis. Comparisons between two groups were conducted using two-tailed *t* test, whereas comparisons among multiple groups were performed using a one-way analysis of variance (ANOVA) followed by appropriate post hoc tests. A value of *p* < 0.05 was considered statistically significant.

## 3. Results

### 3.1. Preparation and Optimization of H007-PC

H007-PC was prepared by solvent evaporation method (Section 2.2.1), and its yield was calculated to be 94.4%.

#### 3.1.1. Infrared Spectral Characterization

**Figure 2:**
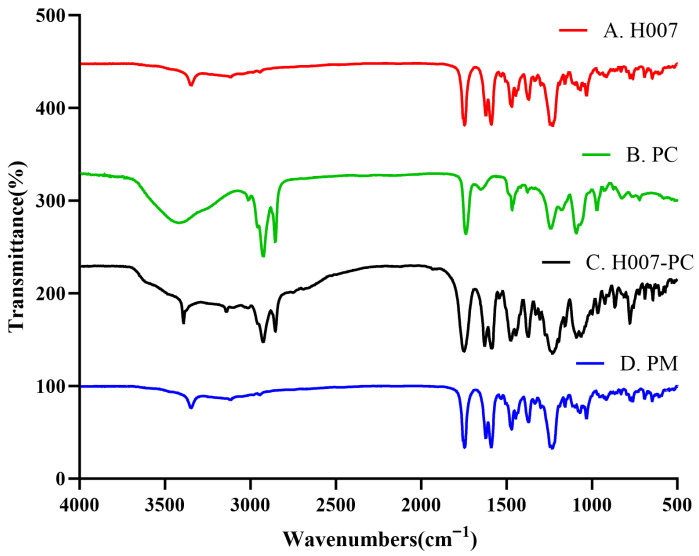
FT-IR spectra of (A) H007, (B) PC, (C) H007-PC and (D) PM.

FT-IR spectra of H007, PC, H007-PC, and their PM were recorded over the wavenumber range of 4000–500 cm^−1^. As shown in Figure 2A, the absorption band at 3347 cm^−1^ in the H007 spectrum corresponds to the stretching vibration of the phenolic hydroxyl group. In Figure 2B, the band at 2853 cm^−1^ is attributed to the C–H stretching vibration of saturated aliphatic chains in PC, whereas the bands at 1737 cm^−1^, 1239 cm^−1^, 1091 cm^−1^ and 766 cm^−1^ correspond to the C=O, P=O, C-O and P-O stretching vibrations, respectively.

Figure 2D shows the FT-IR spectrum of the PM, which represents a simple superposition of the characteristic peaks of H007 and PC, with no obvious shifts in peak position. In contrast, in Figure 2C, the –OH stretching band of H007-PC exhibited a blue shift from 3347 cm^−1^ to 3390 cm^−1^, the -P=O and P-O stretching vibration frequencies also exhibit blue shifts from 1243 cm^−1^ and 775 cm^−1^ to 1271 cm^−1^ and 777 cm^−1^, and the latter is stronger, relative to the PM. These spectral changes (e.g., blue shifts in the –OH and P=O bands) are consistent with the occurrence of intermolecular interactions, such as hydrogen bonding or van der Waals forces [34], between the hydroxyl group of H007 and the polar head groups of PC. No new absorption bands were observed in either the PM or H007-PC spectra, indicating that complex formation occurred without the formation of new covalent bonds.

#### 3.1.2. Differential Scanning Calorimetry Characterization

**Figure 3:**
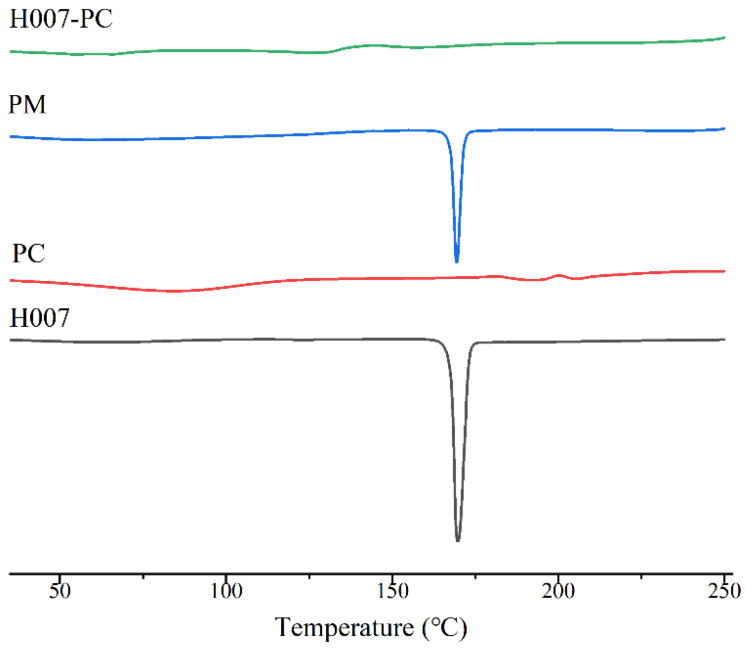
DSC thermograms of H007, PC, H007-PC and PM.

DSC is an effective technique for evaluating interactions between drugs and phospholipids. The DSC thermograms of H007, PC, H007-PC, and PM are shown in Figure 3. Pure H007 exhibited a single endothermic melting peak at approximately 170.5 °C, consistent with its crystalline nature. Conversely, this characteristic melting peak was absent in the H007-PC thermogram. This absence suggests that the ordered crystalline packing of H007 may have been disrupted, possibly by noncovalent interactions such as hydrogen bonding with PC. Together with the IR data, these findings support the successful formation of an amorphous H007-PC complex.

#### 3.1.3. X-Ray Powder Diffraction Characterization

**Figure 4:**
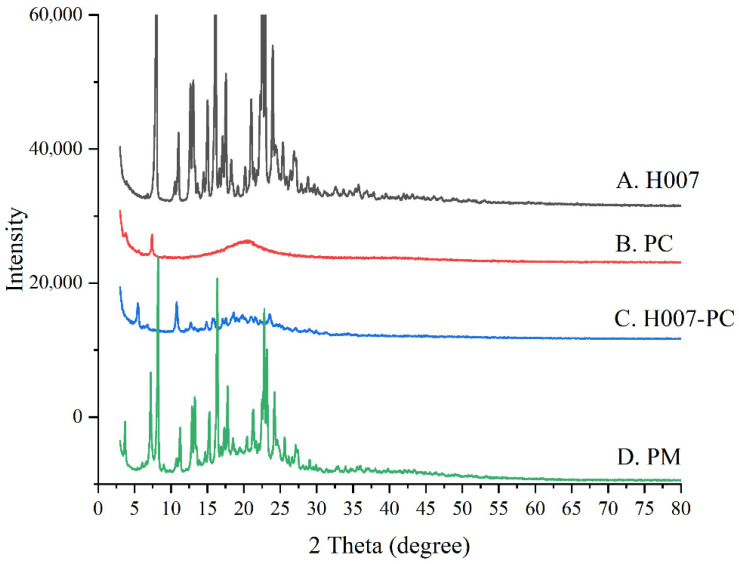
XRPD patterns of H007, PC, H007-PC and PM.

XRPD patterns of H007, PC, H007-PC, and PM are presented in Figure 4. H007 displayed sharp diffraction peaks characteristic of a crystalline structure, whereas PC showed a typical amorphous pattern. In the PM, both the crystalline peaks of H007 and the amorphous features of PC were retained, with no additional diffraction peaks, indicating the absence of solid-state interactions. In contrast, the characteristic crystalline peaks of H007 disappeared in the H007-PC pattern, which exhibited an amorphous profile similar to that of PC, confirming the amorphization of H007 complex formation.

### 3.2. Screening of Excipients for SMEDDS

**Figure 5:**
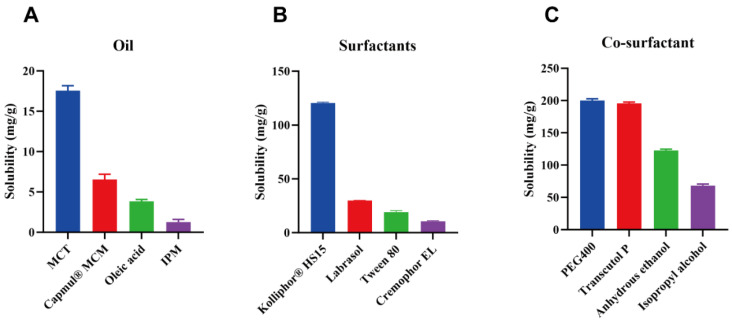
Solubility of H007-PC in various excipients. (**A**) Oils, (**B**) surfactants, and (**C**) co-surfactants. Data are expressed as mean ± SD (*n* = 3).

To develop an SMEDDS capable of dissolving the unit dose of H007 with minimal formulation volume, suitable excipients were first selected according to their solubilization capacity. The solubility of H007-PC in various excipients is summarized in Figure 5. Among the tested oils, MCT showed markedly higher solubility for H007-PC (17.13 mg/g) than the other oils tested (1.04–6.08 mg/g). Solubility in the oil phase is a critical determinant of SMEDDS performance because oils enhance the solubilization of hydrophobic drugs and increase apparent lipophilicity [35]. Therefore, MCT was selected as the oil phase for the SMEDDS formulation.

Kolliphor^®^ HS15 was selected as the surfactant on the basis of systematic excipient screening. Although relatively high solubility values for H007-PC were also observed in polyethylene glycol 400 (198.17 mg/g) and Transcutol P (194.08 mg/g), However, during the experimental investigation, both solvents were found to exhibit poor solubility toward PC. Further evaluation identified anhydrous ethanol as the only solvent capable of dissolving both H007 and PC simultaneously, supporting its selection as the co-surfactant. To increase drug loading capacity, a ternary excipient system consisting of MCT, Kolliphor^®^ HS15, and anhydrous ethanol was designed on the basis of phase compatibility assessment.

### 3.3. Pseudo-Ternary Phase Diagram

**Figure 6:**
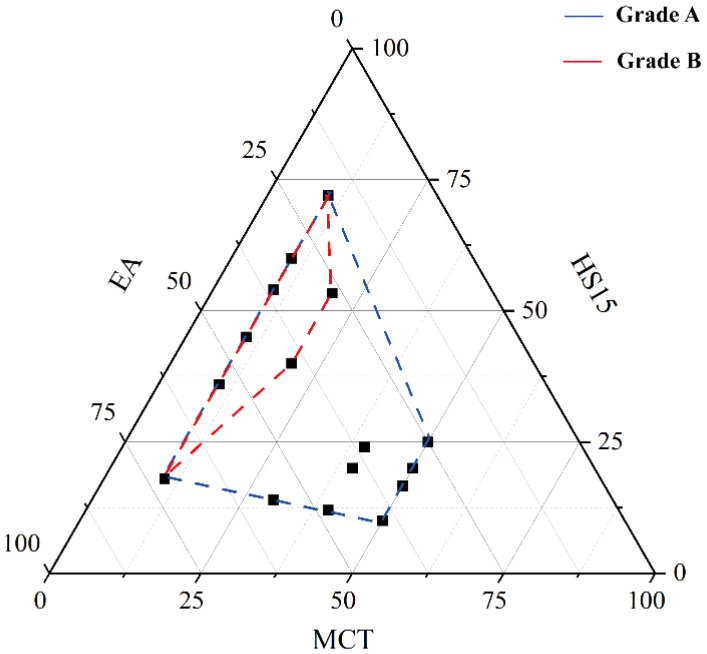
Pseudo-ternary phase diagram consisting of MCT, Kolliphor^®^ HS15, and anhydrous ethanol. The red and blue lines indicate grade A and B microemulsions, respectively.

The self-microemulsifying regions obtained from pseudo-ternary phase diagrams at different Km values showed clear differences, as illustrated in Figure 6. When Km ≥ 4, the self-emulsification time increased markedly, and visible gel-like regions appeared during agitation, which hindered effective drug dispersion. To achieve rapid self-emulsification together with adequate kinetic behavior and formulation stability, SMEDDS generally require only a limited amount of emulsifier. Accordingly, the appropriate Km range was defined as 1–4. Additionally, experimental observations indicated that microemulsion formation was hindered and dispersion transparency decreased when the oil phase concentration exceeded 20%. Therefore, the oil phase content was restricted to 10–20%.

### 3.4. Optimization of H007-PC-SME

The results obtained using the CCD-based optimization of H007-PC-SME are summarized in Table 4. Experimental data were analyzed using Design Expert 13.0 software (Stat-Ease Inc., Minneapolis, MN, USA), and three-dimensional response surface plots were generated. Quadratic polynomial equations were used to describe the relationships between the formulation variables and the response parameters, indicated as follows:

Y_1_ = 34.5 + 4.38*X*_1_ + 1.08*X*_2_ − 6.71*X*_1_*X*_2_ + 1.06*X*_1_^2^ + 1.68*X*_2_^2^ − 7.27*X*_1_^2^*X*_2_ + 6.03*X*_1_*X*_2_^2^ (*R*^2^ = 0.9501, *P* = 0.0055)

Y_2_ = 0.0828 − 0.0414*X*_1_ + 0.0318*X*_2_ − 0.0265*X*_1_
*X*_2_ + 0.0604*X*_1_^2^ + 0.0311*X*_2_^2^ − 0.0896*X*_1_^2^
*X*_2_ + 0.0334*X*_1_
*X*_2_^2^ (*R*^2^ = 0.9488, *P* = 0.0059)

Y_3_ = 81.09 − 3.12*X*_1_ + 0.6081*X*_2_ − 0.9194*X*_1_
*X*_2_ + 2.36*X*_1_^2^ + 0.9370*X*_2_^2^ − 1.11*X*_1_^2^*X*_2_ + 3.8*X*_1_
*X*_2_^2^ (*R*^2^ = 0.9613, *P* = 0.003)

The statistical models and parameters corresponding to the predicted responses, which are derived from the interaction of each factor, are presented in Table 5. Specifically, the statistical models of Y_1_, Y_2_ and Y_3_ are all cubic models. To assess the goodness of fit of these statistical models, several key statistical parameters were adopted, including *p*-value, lack of fit, coefficient of determination (*R*^2^), adjusted *R*^2^ and predicted *R*^2^ [19]. The sequential *p*-value of each model is less than 0.05, indicating that the proposed model is statistically significant at a 95% confidence level. The *R*^2^ and adjusted *R*^2^ values are critical indicators reflecting the degree of consistency between the model-predicted responses and the actual experimental data. In addition, the difference between the *R*^2^ and the adjusted *R*^2^ for all responses is less than 0.2, which further confirms the rationality and reliability of the established models.

The coefficient of determination (*R*^2^) and the corresponding *p*-value were used to evaluate model adequacy. *R*^2^ reflects the agreement between predicted values and experimental observations, whereas *p* < 0.05 indicates statistical significance at the 95% confidence level [36]. Analysis of variance showed that all *R*^2^ values exceeded 0.9, and that the models for Y_1_, Y_2_, and Y_3_ were statistically significant (*p* < 0.05). These results confirm that the relationships between the dependent variables (Y: solubility, droplet size, and PDI) and the independent variables (X: oil weight percentage and Km) were well described by quadratic polynomial models.

**Figure 7:**
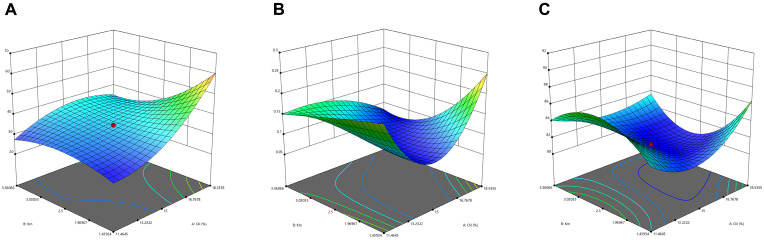
Three-dimensional response surface plots of (**A**) Y_1_: droplet size, (**B**) Y_2_: PDI, (**C**) Y_3_: solubility.

As shown in the three-dimensional response surface plots (Figure 7), increasing the oil phase proportion led to increases in droplet size and PDI. At a fixed oil content, droplet size first increased and then decreased as Km increased. When Km < 3, the average droplet size increased monotonically with increasing oil fraction (W_oil_), whereas when Km > 3, droplet size first increased and then decreased as W_oil_ increased. These trends indicate that a higher emulsifier content facilitates microemulsion formation and that increasing Km contributes to a reduction in PDI. Based on the optimization criteria of solubility, droplet size and PDI, the optimal SMEDDS formulation was determined via multi-objective tradeoff, consisting of 11% (*w*/*v*) MCT as the oil phase, 65% (*w*/*v*) Kolliphor^®^ HS15 as the surfactant, and 24% (*w*/*v*) anhydrous ethanol as the co-surfactant. The model-predicted solubility was 81.09 mg/g, with a droplet size of 20.5 nm and a PDI of 0.267.

To assess predictive accuracy, the percentage error between predicted and experimental values for each response was calculated using the following formula: percentage error = [(observed value − predicted value)/predicted value] × 100 [37]. A percentage error below 10% is generally considered to indicate reliable model performance. As shown in Table 6, all response errors ranged from 0.9% to 8.2%, confirming the robustness and predictive reliability of the statistical model. Therefore, the experimental design successfully optimized the H007-PC-SME, and the selected optimal composition was used in subsequent studies.

### 3.5. Self-Emulsification Performance and TEM Analysis

The self-emulsification behavior of H007-PC-SME was evaluated after dilution in aqueous media. The formulation rapidly formed a homogeneous microemulsion within 60 s, producing a clear, slightly yellow dispersion. No phase separation or drug precipitation was observed within 24 h, indicating good physical stability.

**Figure 8:**
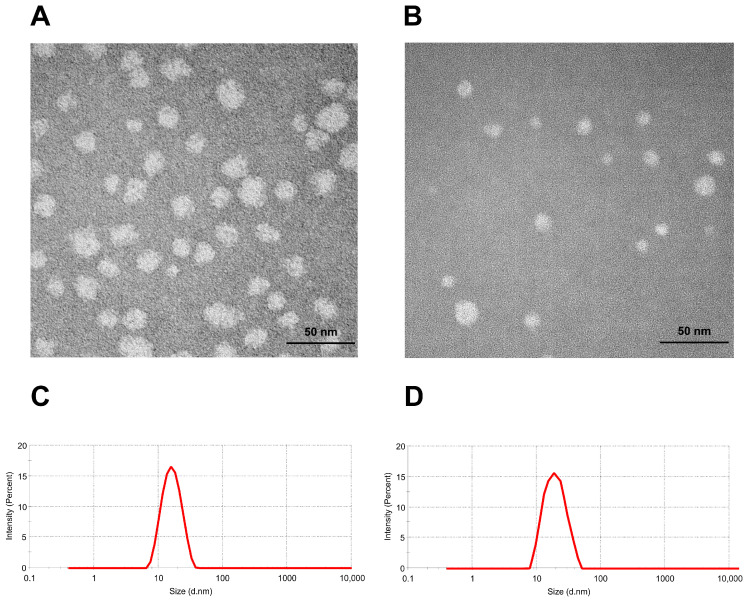
(**A**) TEM images of H007-SME and (**B**) H007-PC-SME. (**C**) Size distribution of H007-SME in water. (**D**) Size distribution of H007-PC-SME in water. **Abbreviations:** H007-SME, conventional H007 self-microemulsions; H007-PC-SME, H007–phospholipid complex self-microemulsions.

TEM images of H007-SME and H007-PC-SME are shown in Figure 8A and Figure 8B, respectively. Both formulations displayed spherical and uniformly distributed microemulsion droplets with comparable morphology and structural characteristics, indicating that incorporation of the phospholipid complex did not substantially alter droplet shape or size distribution. The droplet size in the TEM image was consistent with the droplet size measured by DLS.

### 3.6. Droplet Size and Zeta Potential

In SMEDDS, droplet size is a critical determinant of formulation performance because it directly affects drug release behavior and oral bioavailability. Smaller droplets provide a larger interfacial surface area in contact with the dissolution medium, thereby enhancing dissolution and accelerating drug release. This inverse relationship between droplet diameter and dissolution efficiency provides a theoretical basis for formulation optimization.

Zeta potential is another key parameter in SMEDDS characterization, as it quantitatively reflects the surface charge of emulsion droplets. Surface charge governs colloidal stability by generating electrostatic repulsion between dispersed droplets. Sufficient repulsive forces prevent droplet aggregation and maintain dispersion homogeneity, whereas reduced surface charge may lead to aggregation and phase separation, resulting in instability [38].

As summarized in Table 7, droplet size, zeta potential and PDI were measured to characterize size distribution. The mean droplet diameters of H007-SME (Figure 8C) and H007-PC-SME (Figure 8D) were 20.18 ± 2.13 nm and 20.54 ± 4.23 nm, respectively. There is no significant difference in the zeta potential between H007-SME and H007-PC-SME; both formulations exhibit negative values with absolute magnitudes exceeding 20 mV. The high surface charge contributes to the maintenance of physical stability, indicating that both systems possess favorable physical stability characteristics.

### 3.7. Effect of pH and Dilution Stability

**Figure 9:**
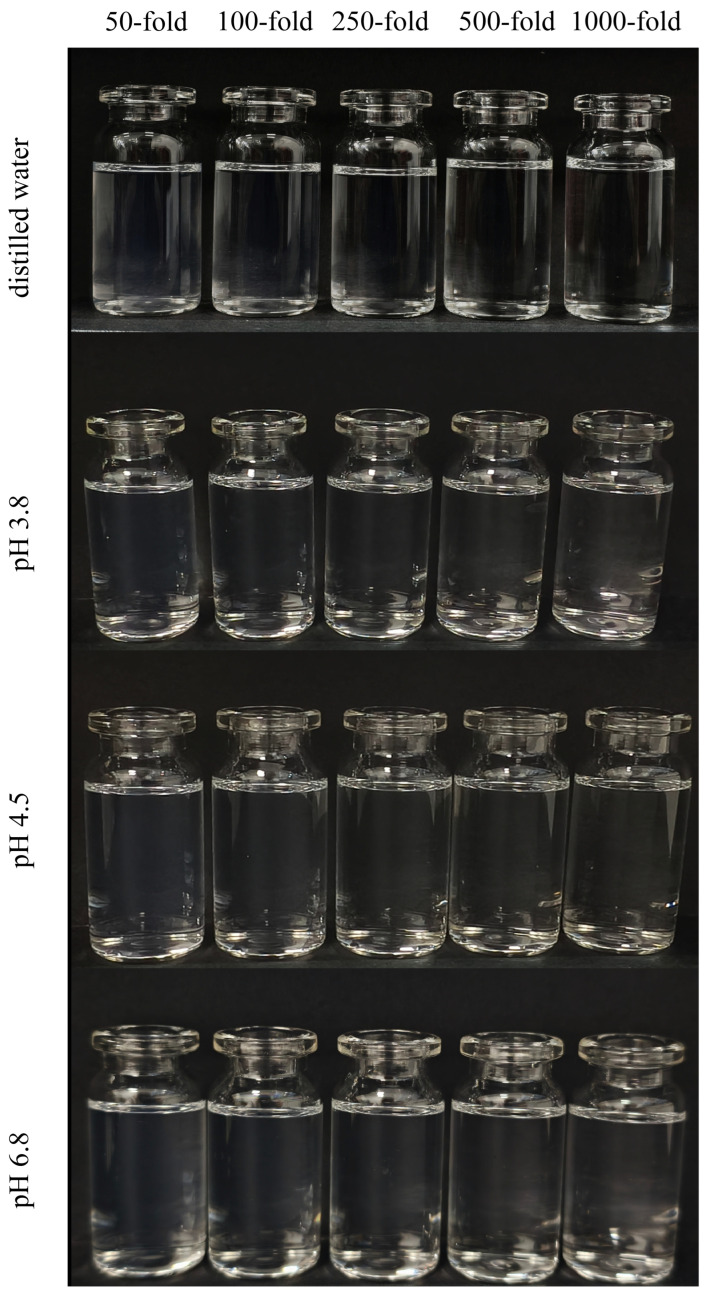
Photographs of the SMEEDS samples after dilution in distilled water, pH 3.8, pH 4.5 and pH 6.8.

After oral administration, SMEDDS undergo progressive dilution upon exposure to gastrointestinal fluids. To evaluate dilution stability, the formulation was diluted 50-fold, 100-fold, 250-fold, 500-fold and 1000-fold with distilled water and buffer solutions at pH 3.8, 4.5, and 6.8. Under all tested conditions, the emulsions remained clear and transparent, with no evidence of drug precipitation or phase separation (Figure 9). These observations demonstrate good dilution and tolerance across a physiologically relevant pH range and indicate that the formulation can maintain dispersion stability during gastrointestinal transit, thereby supporting consistent drug release in vivo.

### 3.8. Stability Test

**Figure 10:**
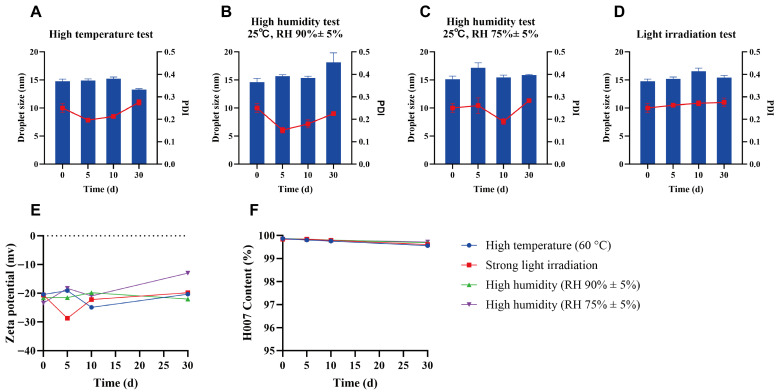
Results of droplet size, PDI, zeta potential and H007 content under stress conditions. (**A**) Elevated temperature test (60 °C); (**B**,**C**) high humidity tests (25 °C, RH 90% ± 5%; 25 °C, RH 75% ± 5%); (**D**) strong light irradiation test (4500 ± 500 lx); (**E**) Zeta potential change curve. (*n* = 3); (**F**) H007 content change curve.

The stability of H007-PC-SME was examined under stress conditions. Changes in droplet size, PDI, zeta potential and H007 content are shown in Figure 10. No meaningful variation in these parameters was observed under most test conditions, indicating good physicochemical stability over the 30-day experimental period. Furthermore, no evidence of drug crystallization was observed during the stability study, based on visual inspection and consistent dispersion characteristics.

However, under elevated temperature conditions, the appearance of the SME began to darken by day 5 and gradually turned dark brown with prolonged exposure. Based on an analysis of H007 content variations in stability test samples, H007 is not regarded as the primary factor responsible for discoloration. As reported [39], lecithin (a component of PC) is susceptible to oxidation and discoloration under elevated temperature conditions. Therefore, the observed discoloration is most likely attributable to phospholipid oxidation, although further mechanistic studies would be required for definitive confirmation. Such discoloration serves as an indicator of compromised stability, directly reflecting oxidative degradation of PC and consequently affecting the overall pharmaceutical performance of the formulation. The observed color change signifies thermal instability and implies that the formulation should be protected from excessive heat exposure.

No obvious discoloration was observed under the other test conditions. Preliminary stability data indicate that the physicochemical properties of H007-PC-SME are retained for at least one month under ambient storage. Nonetheless, formal long-term stability studies are still required to fully characterize its shelf-life profile.

### 3.9. In Vitro Drug Release

**Figure 11:**
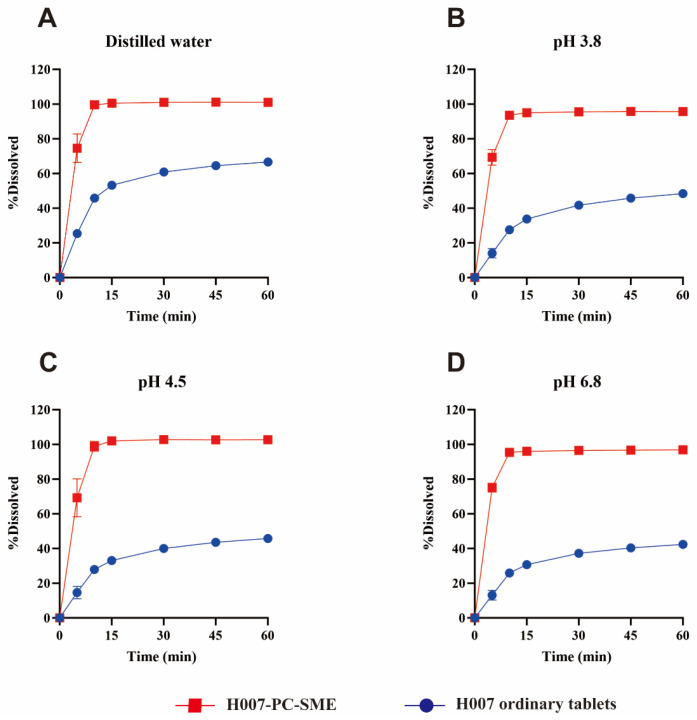
Dissolution profiles of ordinary H007 tablets and H007-PC-SME in the tested media. (**A**) Distilled water; (**B**) pH 3.8 buffer solution; (**C**) pH 4.5 buffer solution; (**D**) pH 6.8 buffer solution. Data are expressed as mean ± SD (*n* = 6).

As shown in Figure 11, dissolution of H007 from ordinary tablets was limited in all tested media. Within 1 h, cumulative release reached only 66%, 48%, 45%, and 42% in distilled water and in buffer solutions at pH 3.8, 4.5 and 6.8, respectively. In contrast, H007-PC-SME markedly improved dissolution behavior, with more than 60% of H007 released within 5 min and more than 90% released within 10 min in all media. The combined use of SMEDDS and phospholipid-complex technology increased the dissolution rate by more than 50% within 1 h. Given that H007 is the 100 mg tablet, while H007-PC-SME is the 30 mg capsule, the paddle method and basket method were utilized separately, with concomitant adjustments to the dissolution medium volume. This experimental protocol was designed to ensure comparability of dissolution profiles under the theoretical condition of 100% drug release. Although the inherent limitations of these dissolution tests should be fully recognized, the comparative analysis strategy based on cumulative percentage release effectively minimized the confounding effects of dosage strength discrepancies on the experimental results. These results indicate that the two formulation strategies act synergistically to improve drug solubility and release performance.

### 3.10. Cell Studies

#### 3.10.1. Cytotoxicity Study

**Figure 12:**
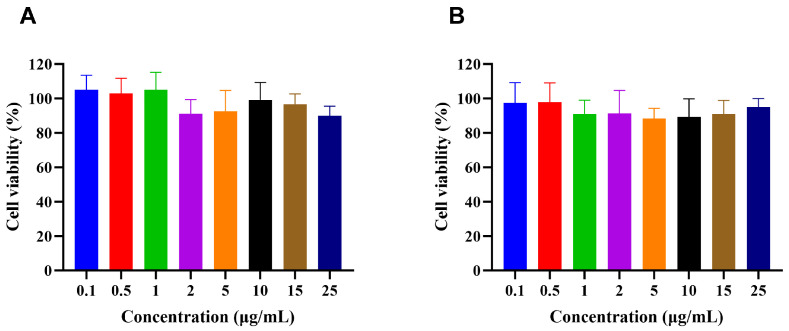
In vitro cytotoxicity on Caco-2 cells for 24 h. (**A**) In vitro cytotoxicity of H007. (**B**) In vitro cytotoxicity of H007-PC-SME. Data are expressed as mean ± SD (*n* = 8).

Cytotoxicity toward Caco-2 cells was evaluated to assess formulation safety. Caco-2 cells differentiate into polarized monolayers with tight junctions and transporter expression and are widely used as an in vitro model of the intestinal epithelium. Cell viability above 70% is generally considered non-toxic, whereas viability below 50% indicates potential irritancy [40]. As shown in Figure 12A, all H007 formulations exhibited concentration-dependent cytotoxicity. H007-PC-SME (Figure 12B) showed a cytotoxicity profile comparable to that of bulk H007. The IC_50_ values of H007 and H007-PC-SME were 0.04879 μg/mL and 0.2729 μg/mL, respectively, with no statistically significant difference (*p* > 0.05). The excipients, including MCT, Kolliphor^®^ HS15, and anhydrous ethanol, are widely used in food, cosmetic, and pharmaceutical products and are generally regarded as safe, which is consistent with the present findings.

These results demonstrate acceptable biocompatibility of H007 and H007-PC-SME within the tested concentration range of 0.1–25 μg/mL. Nonetheless, cytotoxicity data alone are insufficient to support conclusions regarding in vivo safety. Accordingly, while the formulation demonstrates favorable in vitro biocompatibility in this model, further in vivo toxicological studies are warranted to comprehensively assess its overall biological safety profile.

#### 3.10.2. Cellular Uptake Study

Cou-6 was used as a fluorescent surrogate for H007 to evaluate cellular uptake and was encapsulated into the H007-SME and H007-PC-SME formulations. Three groups were examined: free Cou-6, Cou-6-loaded SME (C6-SME), and phosphatidylcholine-modified SME (C6-PC-SME). As shown in Figure 12A, confocal microscopy images obtained after 4 h of incubation illustrate intracellular Cou-6 distribution, with blue fluorescence indicating DAPI-stained nuclei and green fluorescence representing Cou-6.

The free Cou-6 group exhibited minimal green fluorescence, indicating poor cellular uptake. The C6-SME group showed slightly enhanced but still limited uptake. In contrast, the C6-PC-SME group displayed strong and widespread green fluorescence, demonstrating a marked increase in intracellular accumulation after phospholipid modification.

**Figure 13:**
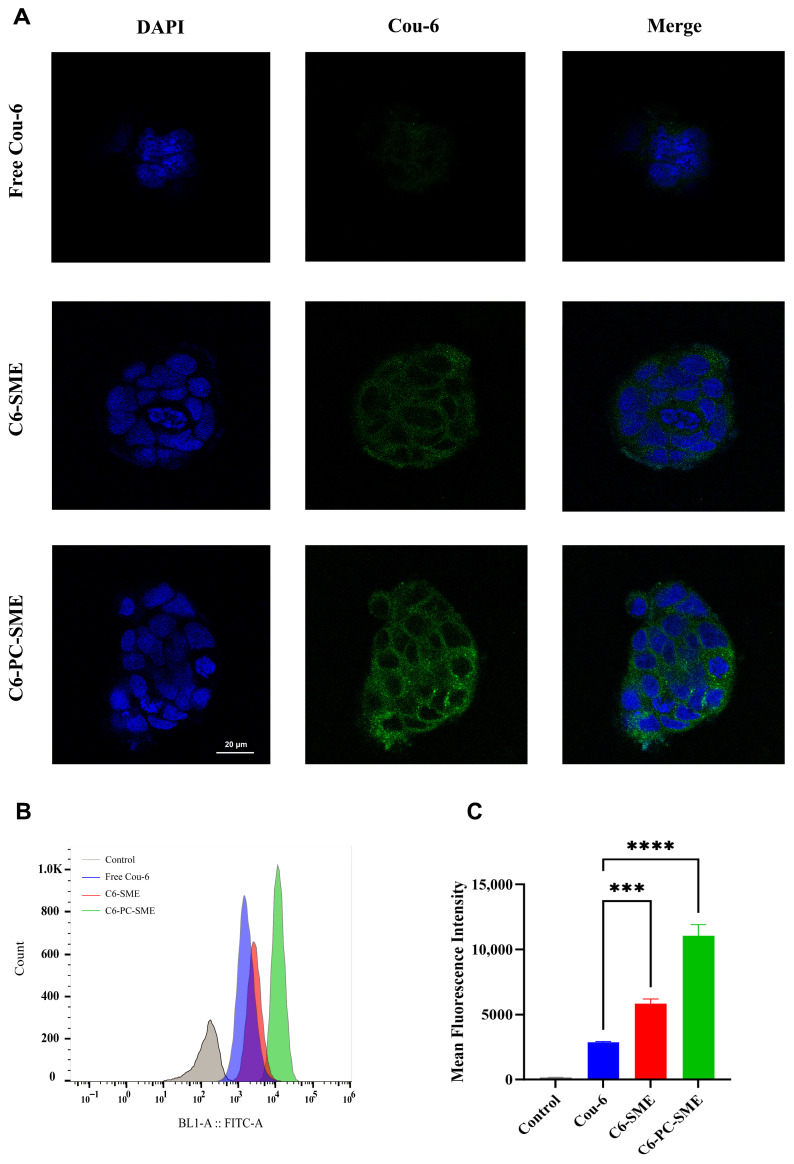
Cellular uptake by Caco-2 cells. (**A**) Confocal images of cellular uptake of different preparations at 4 h. Green: Cou-6; blue: DAPI (nuclei). Analysis of cellular uptake of different preparations using mean fluorescence intensity (**B**) and flow cytometry (**C**). Data are expressed as mean ± SD (*n* = 3). *** *p* < 0.001 and **** *p* < 0.0001.

Quantitative FCM analysis confirmed these observations. Mean fluorescence intensity measurements indicated that cellular uptake of C6-PC-SME was approximately 3.9-fold higher than that of free Cou-6 and approximately 1.8-fold higher than that of C6-SME (Figure 13B,C). These results indicate that phospholipid modification significantly enhances nanoparticle internalization by Caco-2 cells. This enhancement could be attributed to several factors associated with the SMEDDS formulation: the nanoscale droplet structure provides a large surface area for cell membrane interaction, and the phospholipid along with lipophilic excipients may improve membrane affinity and absorption [41]. This strategy appears effective in addressing the limited gastrointestinal permeability of H007.

### 3.11. Pharmacokinetic Study

Previous studies in our laboratory [18] have demonstrated that H007 acts as a prodrug and that its metabolic conversion does not depend on the cytochrome P450 system. Instead, H007 is rapidly hydrolyzed and deacetylated by esterases in plasma and tissues to generate the metabolite M1. M1 is subsequently phosphorylated by adenosine kinase to form MP, the active metabolite of H007. Therefore, M1 and MP were selected as surrogate markers for evaluating oral bioavailability, and pharmacokinetic parameters were calculated on the basis of their plasma concentrations.

After a single oral dose, H007 was rapidly absorbed in both male and female golden hamsters. Metabolites M1 and MP were detectable in plasma within 5 min after administration in all treatment groups. Plasma concentrations of M1 and MP declined to near the lower limit of quantification by 12 h post-administration. In contrast, plasma concentrations of the parent compound remained close to or below the detection limit at all time points.

**Figure 14:**
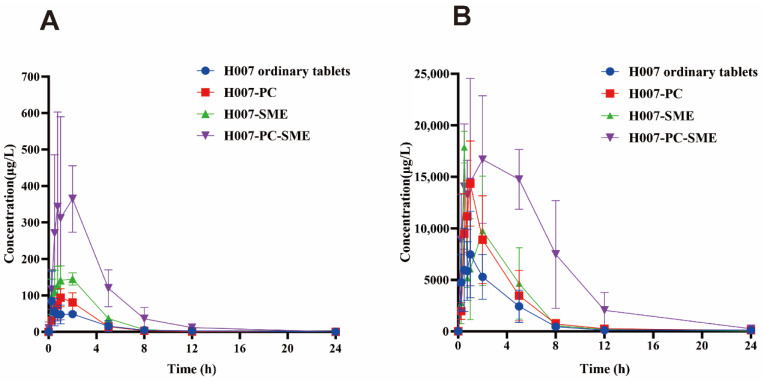
Mean plasma concentration–time curves in golden hamsters after oral administration of H007, H007-PC, H007-SME and H007-PC-SME. (**A**) M1; (**B**) MP. Data are expressed as mean ± SD (*n* = 6). **Abbreviations:** H007-PC, H007–phospholipid complex; H007-SME, conventional H007 self-microemulsions; H007-PC-SME, H007–phospholipid complex self-microemulsions.

Figure 14 shows the plasma concentration–time profiles of M1 and MP following administration of the different formulations. Both H007-SME and H007-PC-SME produced markedly higher plasma concentrations of M1 and MP than ordinary H007 tablets and H007-PC. Relative to ordinary H007 tablets, the C_max_ of M1 increased by approximately 5-fold for H007-SME and 5.3-fold for H007-PC-SME. As summarized in Table 7, the relative bioavailability values based on M1 exposure were 376.65% for H007-SME and 464.62% for H007-PC-SME. In addition, the AUC_0–t_ of M1 for H007-PC-SME was approximately 1.2-fold higher than that for H007-SME. Data from Table 8 and Table 9 consistently indicate the following rank order for plasma concentration, C_max_, and AUC_0–t_ of both M1 and MP: H007-PC-SME > H007-SME > H007-PC > H007. These findings confirm that SMEDDS markedly improves the oral bioavailability of H007 and that incorporation of H007-PC into SMEDDS provides an additional pharmacokinetic advantage over conventional SMEDDS.

Nevertheless, it should be acknowledged that the AUC and C_max_ parameters observed in this study displayed a certain degree of variability, which may be attributed to inherent physiological heterogeneity among the experimental animals. However, following log-transformation of the data and rigorous statistical analysis using ANOVA with appropriate post hoc tests, the inter-group differences were confirmed to be statistically significant. Accordingly, despite the observer variability, the conclusion that H007-PC-SME significantly improves oral bioavailability remains robust and well-supported.

### 3.12. Lymphatic Transport

Lipophilic compounds absorbed by intestinal epithelial cells may enter the systemic circulation through either the portal venous route or the lymphatic pathway. Lymphatic transport is closely associated with intracellular chylomicron synthesis and secretion [42,43,44]. Previous studies have shown that cycloheximide effectively suppresses chylomicron secretion and thereby blocks lymphatic transport, producing an effect functionally comparable to lymphatic cannulation [42,43,44]. However, a cycloheximide model only reflects the overall changes in pharmacokinetics following the inhibition of chylomicron formation and secretion, and it cannot distinguish whether the drug is absorbed by lymphocytes during lymphatic transport or directly conveyed within the lymphatic fluid. Consequently, it may serve primarily as a source of indirect evidence.

Earlier pharmacokinetic investigations in our laboratory have established that H007 is rapidly converted to M1 and MP in plasma. Accordingly, systemic exposure to M1 and MP within 24 h after oral administration was used as an indirect indicator for assessing lymphatic transport.

**Figure 15:**
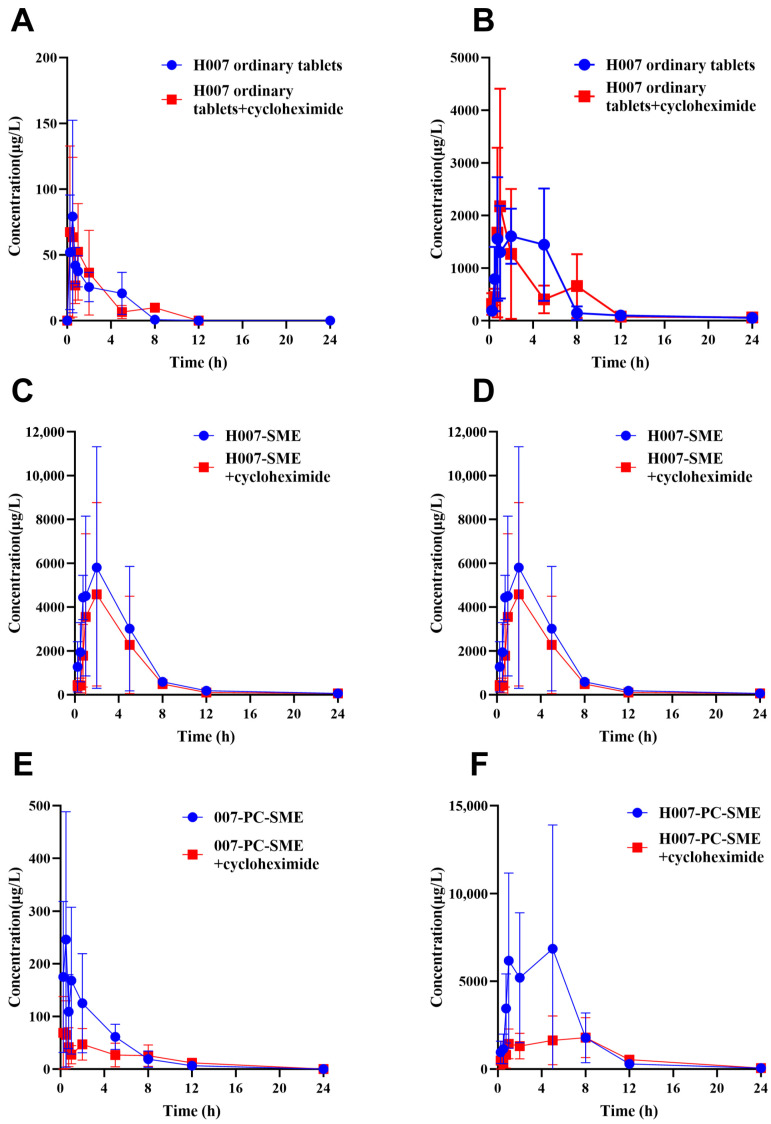
Mean plasma concentration–time curves of M1 and MP in golden hamsters within 24 h after intraperitoneal pretreatment with or without 5.0 mg/kg cycloheximide followed by oral administration of H007, H007-SME and H007-PC-SME. **Notes:** Panels (**A**,**C**,**E**) show M1 profiles, and panels (**B**,**D**,**F**) intraperitoneal pretreatment with or without 5.0 mg/kg cycloheximide following oral administration of show MP profiles. Data are expressed as mean ± SD (*n* = 3). **Abbreviations:** H007-SME, conventional H007 self-microemulsions; H007-PC-SME, H007–phospholipid complex self-microemulsions.

As shown in Figure 15, pretreatment with cycloheximide significantly reduced the mean plasma concentrations of M1 and MP after administration of H007-SME and H007-PC-SME, whereas plasma concentrations after administration of ordinary H007 tablets were not significantly affected. This finding indicates that lymphatic transport contributes minimally to systemic exposure after administration of ordinary H007 tablets. Notably, inhibition of lymphatic transport caused a greater reduction in AUC_0–t_ for H007-PC-SME than for H007-SME. Nevertheless, the AUC_0–t_ values of both formulations remained higher than those of ordinary H007 tablets. These results indicate that SMEDDS formulations containing H007-PC enhance oral bioavailability not only by promoting lymphatic transport but also through additional absorption-enhancing mechanisms.

Specifically, the AUC_0–t_ values of M1 and MP presented in Table 10 and Table 11 represent total systemic exposure derived from all absorption routes. After cycloheximide pretreatment, the AUC_0–t_ values shown in Table 12 and Table 13 represent exposure attributable solely to non-lymphatic absorption pathways. Therefore, based on a comprehensive analysis of metabolite exposure and the cycloheximide model, the approximate fraction transported via the lymphatic system was calculated as the difference in AUC_0–t_ between untreated and cycloheximide-pretreated animals. The calculated lymphatic fractions are summarized in Table 14 and Table 15.

Pharmacokinetic analysis (Table 14 and Table 15) showed that, relative to ordinary H007 tablets, H007-SME and H007-PC-SME increased the lymphatic transport fraction of M1 from 0% to 22% and 54%, respectively, and increased the lymphatic transport fraction of MP from 1% to 28% and 52%, respectively. Based on the calculated AUC differences, the estimated contribution of lymphatic transport to systemic exposure followed the order: H007-PC-SME > H007-SME > H007. Given the inherent limitations of the cycloheximide model, these findings should be viewed as indicative of the lymphatic transport propensity of H007-PC-SME, rather than providing a precise quantification of its absolute contribution.

## 4. Conclusions

This study addressed the challenges posed by the poor aqueous solubility and limited membrane permeability of H007 through the development of H007-PC-SME. H007-PC was successfully prepared by a solvent-evaporation method. The results demonstrated that SMEDDS markedly improves the solubility and gastrointestinal absorption of H007 through a simple and scalable formulation strategy. By integrating phospholipid-complex formation with SMEDDS technology, the H007-PC-SME formulation was systematically developed and optimized using experimental design.

Compared with ordinary H007 tablets, H007-PC-SME substantially enhanced H007 solubility in multiple dissolution media and significantly accelerated drug release, reflecting the synergistic effect of the combined formulation strategies. Cellular uptake studies further confirmed enhanced permeability, indicating that this approach improves key biopharmaceutical properties and may translate into improved therapeutic performance.

Pharmacokinetic evaluation showed that H007-PC-SME markedly increases oral bioavailability. In addition, results from the chylomicron flow blockade model indicate that oral administration of H007-PC-SME promotes lymphatic transport, supporting its potential relevance for lymphatic-targeted drug delivery. Overall, these findings demonstrate that H007-PC-SME effectively overcomes the intrinsic limitations of H007 at both the in vitro and in vivo levels and represents a promising strategy for further pharmaceutical development.

## Figures and Tables

**Table 1:** Classification standards of emulsification grade.

Grade	Emulsification Time	Appearance
I	<1 min	Clear or slightly blue
II	<1 min	Bright white emulsion
III	1–2 min	Milky emulsion
IV	>2 min	Grayish white emulsion with a slightly oil
V	Difficult to emulsify	Large oil droplets presenting

**Table 2:** Factors and levels of the central composite design.

Factors	Level				
	−1.414	−1	0	1	1.414
X_1_	10	11.46	15	18.54	20
X_2_	1	1.44	2.5	3.56	4

**Table 3:** The procedure of gradient elution.

Time (min)	Mobile Phase A	Mobile Phase B
0.00	95	5
0.50	95	5
0.60	65	5
2.00	65	5
2.01	95	5
4.00	95	5

**Table 4:** The composition of H007-PC-SME and observed responses through central point design (*n* = 3).

Run	Factors		Responses		
	X_1_	X_2_	Y_1_	Y_2_	Y_3_
	Oil (%)	Km	Particle Size (nm)	PDI	Solubility (mg·g^−1^)
1	11.46	3.56	29.72	0.17	83.48
2	15	4	37.01	0.171	84.48
3	15	1	33.96	0.081	82.76
4	10	2.5	28.03	0.243	90.88
5	15	2.5	34.44	0.08	81.25
6	18.54	3.56	37.12	0.101	82.98
7	15	2.5	34.22	0.078	81.21
8	15	2.5	34.27	0.078	80.92
9	15	2.5	34.73	0.092	80.97
10	18.54	1.44	62.97	0.27	85.82
11	11.46	1.44	28.7	0.233	82.64
12	15	2.5	34.85	0.086	81.09
13	20	2.5	40.41	0.126	82.05

**Table 5:** Statistical models and parameters of D-optimal mixture design.

Responses	Suggested Model	Model *p*-Value	Lack of Fit *p*-Value	*R* ^2^	Adjusted *R*^2^	Adequate Precision
Y_1_	Cubic	0.0055	<0.0001	0.9501	0.8803	14.4184
Y_2_	Cubic	0.0059	0.0009	0.9488	0.8771	9.2878
Y_3_	Cubic	0.0030	0.0002	0.9613	0.9070	13.8073

**Table 6:** Predicted and observed values of optimal H007-PC-SME (*n* = 3).

Optimal Factors	Responses	Predicted Value	Observed Value	Error Percentage (%)
X_1_: 11%	Y_1_: Particle size (nm)	20.5	20.53 ± 0.45	0.15
X_2_: 2.7	Y_2_: PDI	0.267	0.25 ± 0.02	6.37
	Y_3_: Solubility (mg·g^−1^)	81.09	80.23 ± 1.02	1.06

**Table 7:** Characterization of H007-SME and H007-PC-SME (*n* = 3).

Formulation	H007-SME	H007-PC-SME
Content of H007 (mg/g)	50	50
Efficiency of self-emulsification	I	I
Droplet size (nm)	20.18 ± 2.13	20.54 ± 4.23
Polydispersity index	0.219 ± 0.121	0.247 ± 0.102
Zeta potential (mV)	−20.3 ± 1.8	−20.9 ± 2.2

**Table 8:** Pharmacokinetic parameters of M1 after oral administration of H007, H007-PC, H007-SME and H007-PC-SME *(n* = 6).

Pharmacokinetic Parameters	H007	H007-PC	H007-SME	H007-PC-SME
AUC_0–t_ (µg/L × h)	231.32 ± 38.39	316.57 ± 70.46	871.28 ± 504.31 *	1074.77 ± 204.69 **^,#^
MRT_0–t_ (h)	2.45 ± 0.81	2.23 ± 0.57	2.57 ± 0.57	4.92 ± 1.08
T_max_ (h)	0.75 ± 0.73	1.29 ± 0.56	1.20 ± 0.76	1.50 ± 1.84
C_max_ (ug/L)	97.78 ± 70.89	107.70 ± 16.73	490.22 ± 306.29 *	513.91 ± 228.85 **^,#^
Relative bioavailability (%)	100.00	136.85	376.65	464.62

**Abbreviations:** H007, ordinary H007 tablets; H007-PC, H007–phospholipid complex; H007-SME, conventional H007 self-microemulsions; H007-PC-SME, H007–phospholipid complex self-microemulsions; * *p* < 0.05 and ** *p* < 0.01 compared with H007 and H007-PC; ^#^ *p* < 0.05 compared with H007-SME.

**Table 9:** Pharmacokinetic parameters of MP after oral administration of H007, H007-PC, H007-SME and H007-PC-SME (*n* = 6).

Pharmacokinetic Parameters	H007	H007-PC	H007-SME	H007-PC-SME
AUC_0–t_ (µg/L × h)	29,888.67 ± 5070.45	48,000.32 ± 16,581.27	93,854.77 ± 34,129.66 *	13,8547.84 ± 22,698.32 **^,#^
MRT_0–t_ (h)	3.56 ± 0.57	3.29 ± 0.61	1.87 ± 0.61	5.15 ± 1.10
T_max_ (h)	0.67 ± 0.30	0.95 ± 0.11	0.5	2.40 ± 3.19
C_max_ (µg/L)	9807.38 ± 2660.64	14,440.95 ± 4013.10	17,868.19 ± 1535.63 *	20,429.59 ± 5349.82 **^,#^
Relative bioavailability (%)	100	160.60	314.01	463.55

**Abbreviations:** H007, ordinary H007 tablets; H007-PC, H007–phospholipid complex; H007-SME, conventional H007 self-microemulsions; H007-PC-SME, H007–phospholipid complex self-microemulsions; * *p* < 0.05 and ** *p* < 0.01 compared with H007 and H007-PC; ^#^ *p* < 0.05 compared with H007-SME.

**Table 10:** Pharmacokinetic parameters of M1 after oral administration of H007, H007-SME and H007-PC-SME (*n* = 3).

Pharmacokinetic Parameters	H007	H007-SME	H007-PC-SME
AUC_0–t_ (µg/L × h)	146.78 ± 40.03	552.43 ± 372.10 *	745.89 ± 338.09 **^,#^
MRT_0–t_ (h)	2.02 ± 0.894	2.539 ± 0.46	3.41 ± 1.14
T_max_ (h)	1.50 ± 1.96	0.8 ± 0.74	1.45 ± 2.00
C_max_ (µg/L)	91.62 ± 61.98	187.938 ± 125.68 *	270.65 ± 219.33 **^,#^

**Abbreviations:** H007, ordinary H007 tablets; H007-SME, conventional H007 self-microemulsions; H007-PC-SME, H007–phospholipid complex self-microemulsions; AUC, area under the curve; MRT, mean residence time; T_max_, time to peak concentration; C_max_, maximum concentration. * *p* < 0.05 and ** *p* < 0.01 compared with H007; ^#^ *p* < 0.05 compared with H007-SME.

**Table 11:** Pharmacokinetic parameters of MP after oral administration of H007, H007-SME and H007-PC-SME (*n* = 3).

Pharmacokinetic Parameters	H007	H007-SME	H007-PC-SME
AUC_0–t_ (µg/L × h)	9245.86 ± 4228.67	29,224.44 ± 20,094.33 *	41,491.45 ± 20,855.96 **^,#^
MRT_0-t_ (h)	4.47 ± 1.05	4.24 ± 0.97	4.56 ± 1.54
T_max_ (h)	1.60 ± 1.91	1.92 ± 1.62	3.67 ± 2.81
C_max_ (µg/L)	2750.28 ± 772.95	6831.254 ± 4659.84 *	10,491.61 ± 6897.22 **^,#^

**Abbreviations:** H007, ordinary H007 tablets; H007-SME, conventional H007 self-microemulsions; H007-PC-SME, H007–phospholipid complex self-microemulsions; AUC, area under the curve; MRT, mean residence time; T_max_, time to peak concentration; C_max_, maximum concentration. * *p* < 0.05 and ** *p* < 0.01 compared with H007; ^#^ *p* < 0.05 compared with H007-SME.

**Table 12:** Pharmacokinetic parameters of M1 after intraperitoneal pretreatment with 5.0 mg/kg cycloheximide following oral administration of H007, H007-SME and H007-PC-SME (*n* = 3).

Pharmacokinetic Parameters	H007 + Inhibition	H007-SME + Inhibition	H007-PC-SME + Inhibition
AUC_0–t_ (µg/L × h)	180.56 ± 133.33	432.76 ± 237.69 *	347.413 ± 209.40 **^,#^
MRT0–t (h)	2.42 ± 1.10	3.35 ± 1.46	4.861 ± 0.69
T_max_ (h)	0.6 ± 0.38	2.00 ± 1.64	2.55 ± 2.33
C_max_ (µg/L)	76.43 ± 67.46	109.75 ± 68.06 *	74.788 ± 64.64 **^,#^

**Abbreviations:** H007, ordinary H007 tablets; H007-SME, conventional H007 self-microemulsions; H007-PC-SME, H007–phospholipid complex self-microemulsions; AUC, area under the curve; MRT, mean residence time; T_max_, time to peak concentration; C_max_, maximum concentration. * *p* < 0.05 and ** *p* < 0.01 compared with H007; ^#^ *p* < 0.05 compared with H007-SME.

**Table 13:** Pharmacokinetic parameters of MP after intraperitoneal pretreatment with 5.0 mg/kg cycloheximide followed by oral administration of H007, H007-SME and H007-PC-SME (*n* = 3).

Pharmacokinetic Parameters	H007 + Inhibition	H007-SME + Inhibition	H007-PC-SME + Inhibition
AUC_0-t_ (µg/L × h)	9140.53 ± 5624.56	21057.702 ± 13631.80 *	19802.7 ± 7948.52 **^,#^
MRT_0–t_ (h)	6.07 ± 1.91	5.113 ± 2.08	7.487 ± 1.03
T_max_ (h)	0.95 ± 0.11	3.00 ± 1.87	5.00 ± 2.12
C_max_ (µg/L)	2535.54 ± 2183.99	5889.426 ± 5090.13 *	2222.826 ± 1240.83 **^,#^

**Abbreviations:** H007, ordinary H007 tablets; H007-SME, conventional H007 self-microemulsions; H007-PC-SME, H007–phospholipid complex self-microemulsions; AUC, area under the curve; MRT, mean residence time; T_max_, time to peak concentration; C_max_, maximum concentration. * *p* < 0.05 and ** *p* < 0.01 compared with H007; ^#^ *p* < 0.05 compared with H007-SME.

**Table 14:** Proportion of M1 reaching systemic circulation directly versus via the intestinal lymphatic pathway after oral administration of H007 in different formulations to golden hamsters (*n* = 3).

Formulations	Fraction Transported via Lymphatic System (%)	Fraction Transported Directly to Systemic Circulation (%)
H007	-	123
H007-SME	22	78
H007-PC-SME	54	46

**Abbreviations:** H007, ordinary H007 tablets; H007-SME, conventional H007 self-microemulsions; H007-PC-SME, H007–phospholipid complex self-microemulsions.

**Table 15:** Proportion of MP reaching systemic circulation directly versus via the intestinal lymphatic pathway after oral administration of H007 in different formulations to golden hamsters (*n* = 3).

Formulations	Fraction Transported via Lymphatic System (%)	Fraction Transported Directly to Systemic Circulation (%)
H007	1	99
H007-SME	28	72
H007-PC-SME	52	48

**Abbreviations:** H007, ordinary H007 tablets; H007-SME, conventional H007 self-microemulsions; H007-PC-SME, H007–phospholipid complex self-microemulsions.

## Data Availability

The original contributions presented in this study are included in the article. Further inquiries can be directed to the corresponding authors.
